# Antibacterial effect and impact on caries activity of nanosilver fluoride and silver diamine fluoride in dentin caries of primary teeth: a randomized controlled clinical trial

**DOI:** 10.1186/s12903-022-02697-y

**Published:** 2022-12-30

**Authors:** Nour Ammar, Magda M. El-Tekeya, Sara Essa, Marwa M. Essawy, Dalia M. Talaat

**Affiliations:** 1 Department of Pediatric Dentistry and Dental Public Health, Faculty of Dentistry, Alexandria University, Alexandria, Egypt; 2 Department of Medical Microbiology and Immunology, Faculty of Medicine, Alexandria University, Alexandria, Egypt; 3 Department of Oral Pathology, Faculty of Dentistry, Alexandria University, Alexandria, Egypt; 4 Center of Excellence for Research in Regenerative Medicine and Applications (CERRMA), Faculty of Medicine, Alexandria University, Alexandria, Egypt

**Keywords:** Silver nanoparticles, Silver diamine fluoride, Logistic regression, Dental caries, Streptococci, Primary teeth, Pediatric dentistry, Child, RCT

## Abstract

**Background:**

The use of silver diamine fluoride (SDF) in caries treatment in children has increased despite the disadvantage of causing tooth discoloration. Nanosilver fluoride (NSF) is a possible alternative. This study aimed to assess the antibacterial effect of NSF and SDF and their impact on the activity of dentin caries in primary teeth.

**Methods:**

Synthesis and characterization of the physical and biological properties of NSF were conducted. Fifty children aged 4–6 years with dentin caries (active caries corresponding to ICDAS code 5) in deciduous teeth were randomly assigned to treatment by NSF or SDF. Baseline assessment of *Streptococcus mutans* (*S. mutans*) and *lactobacilli* counts as CFU/mL in caries lesions was done, followed by the application of the agents. After one month, microbiological samples were recollected, and lesion activity was reassessed. Groups were compared using Mann–Whitney and Chi-Square tests, while intragroup comparisons were done using Wilcoxon and McNemar tests. Multilevel logistic regression analysis was used to assess the effect of different variables on the outcomes.

**Results:**

There were 130 teeth in 50 children; mean ± SD age = 4.75 ± 0.76 years, 63% were posterior teeth. At the one-month follow-up appointment, both groups showed a significant decrease from baseline bacterial counts. There was a significant difference in the reduction of *S. mutans* between NSF and SDF (21.3% and 10.5%, respectively, *p* = 0.002), while not in *lactobacilli* (13.9% and 6.0%, respectively, *p* = 0.094). In both groups, there was a significant reduction in the number of active caries from baseline (*p* < 0.0001) with no significant difference between groups (percentage inactive = 64.4% and 63.4%, *p* = 0.903). Multilevel regression revealed non-significant differences in *S. mutans* and *lactobacilli* counts (AOR 1.281, *p* = 0.737 and 1.888, *p* = 0.341, respectively), and in the number of inactive lesions (AOR 1.355, *p* = 0.731) between groups.

**Conclusion:**

The short-term antibacterial efficacy of NSF was similar to that of SDF. In both groups there was a significant reduction of *S. mutans* and *lactobacilli* counts in active dentin caries, and two-thirds of the lesions became inactive with no differences between the two interventions. Further research is needed to investigate the long-term efficacy of NSF and its suitability for clinical use in caries management.

*Trial registration*: This trial was prospectively registered on the clinicaltrials.gov registry with ID: NCT05221749 on 03/02/2022.

## Introduction

The high prevalence of dental caries in children renders it a global priority area for action. The caries process is initiated after an imbalance in the ecology of the biofilm surrounding the tooth. This imbalance favors the growth of cariogenic bacteria which in turn produce demineralizing acids that degenerate dental hard tissues. The amount of the cariogenic bacteria *Streptococcus mutans* (*S. mutans*) and *lactobacilli* increases in the presence of fermentable carbohydrates which fuel the caries process [1].

In recent years, minimally invasive dentistry (MID) has become increasingly adopted by clinicians [2]. MID is a conservative approach that implements individualized caries risk assessment with an enhanced focus on early prevention and interception of the caries process. This biological approach aims to preserve sound tooth tissue as well as tissue that can potentially be remineralized [3]. If the cariogenic challenge of a specific environment is controlled and suitable therapeutic agents are used the caries process can be arrested [4]. Among the multiple MID approaches, silver diamine fluoride (SDF) has been one of the most popular ones [2]. SDF has a proven potent antibacterial effect on cariogenic bacteria and systematic reviews conclude that SDF is safe and effective in arresting caries of the primary dentition. However, the inherent disadvantage of SDF is the black staining of carious dentin and enamel [5].

With the innovation of nanotechnology, new modalities for caries management have been proposed. Silver nanoparticles (AgNPs) have significant antibacterial activity against *S. mutans* in low concentrations [6]. This antibacterial activity has been documented in *in-vitro* and clinical research [7–9]. AgNPs can be combined with fluoride in solution to produce nanosilver fluoride (NSF) which is available as a clear yellowish solution that is economical to produce [10]. AgNPs can be synthesized through several methods, of which the citrate reduction method is commonly used in combination with polyethylene glycol (PEG) coating of the nanoparticles, producing PEG-coated AgNPs (PEG-AgNP) [7, 11].

Contrary to SDF, NSF does not stain the tooth structure [12]. Clinical trials comparing the cariostatic effect of NSF in comparison to SDF have yielded consistent results; NSF has a highly comparable anti-caries efficacy to that of SDF [13, 14]. The combination of these desirable properties has made NSF a candidate for use in MID to overcome the disadvantages of SDF.

Unlike clinical trials comparing the cariostatic potential of NSF and SDF [9, 15, 16], trials comparing the antibacterial efficacy of these agents are scarce. Therefore, this randomized controlled clinical trial aimed to compare the change in counts of *S. mutans* and *lactobacilli* in dentin caries as well as the change in caries activity after one month of treatment with NSF or SDF. The null hypotheses of this study were that after one month of treatment with either NSF or SDF, there would be no differences neither in the change in counts of *S. mutans* and *lactobacilli* nor in the decrease in the number of active caries lesions between groups.

## Materials and methods

### PEGylated nanosilver preparation with physical and biological characterization

Polyethylene glycol (PEG 400) coated AgNPs were prepared following the citrate reduction method [11, 17]. Preliminary characterization of the nanoparticles was conducted using UV–visible spectrophotometer (Nanodrop, DeNovix, DS-11 FX+, US), where the absorbance spectra wavelength was scanned in the 200–800 nm range. The average particle size, polydispersity index (PDI), and surface charge of AgNPs were detected by Zeta-seizer (Nano ZS, Malvern Instruments, Worcestershire, UK). The nanoparticles were also characterized using transmission electron microscopy (TEM) (JEOL JEM-1400 series 120 kV TEM).

Human gingival fibroblasts and oral squamous cell carcinoma 4 cell line (SCC 4, American Type Culture Collection (ATCC), VA, USA) were used to certify the safety and determine the selectivity index of the PEG-AgNPs. The human gingival fibroblasts were isolated by the Center of Excellence for Research in Regenerative Medicine and its Application (CERRMA) at the Faculty of Medicine, Alexandria University after obtaining informed consent from all donors. Following the protocol of an MTT assay, the cytotoxic effect of nanoparticles on SCC 4 and gingival fibroblasts was evaluated. We followed the method described in our published protocol to determine the minimum inhibitory concentration (MIC) of PEG-AgNPs on the reference strain of *S. mutans* [17].

After determining the cellular IC_50_ from the MTT assay and the MIC, 10 mL of 5% sodium fluoride (22,600 ppm) were added to the PEG-AgNP solution in a lightproof brown bottle, achieving an equivalent concentration of 256 μg/mL PEG-AgNPs. The solution was stirred overnight to achieve uniform dispersion of the particles.

### Clinical trial

#### Ethical considerations and design

This trial was prospectively registered on the clinicaltrials.gov registry (#NCT05221749). This parallel, two-arm, randomized controlled clinical trial was conducted in the Department of Pediatric Dentistry at the Faculty of Dentistry, Alexandria University, Egypt. Ethical approval was granted by the ethics committee at the faculty (#0359-12/2021). Participants were provided with treatment for teeth showing failure after the intervention. The PICOT question was: will 4–6 years old children with an active carious lesion have a greater decrease in cariogenic bacteria counts and active caries lesions after one month of using NSF than SDF?

#### Sample size estimation

Sample size was based on estimates reported by Dos Santos et al. [9] and Milgrom et al. [18] where the percentage of arrested decay was 14.54% and 51.7% for NSF and 38% SDF treated teeth, respectively. To achieve 80% power with α = 0.05, 24 patients per group were required, this number was increased to 25 patients to make up for loss to follow. The total sample size for the two groups was 50 patients as calculated by G*power 3.1.9.7.

#### Participant eligibility and examination

Participants were included if they were: children 4–6 years old with at least one active carious lesion on a primary tooth corresponding to code 5 of the International Detection and Assessment System (ICDAS). This is characterized by a distinct cavity with visible dentine, not including more than half of the tooth [19]. The completion of the informed consent was a prerequisite to participation. The exclusion criteria were: teeth causing spontaneous or elicited pain, with signs of pulpal infection, or prematurely mobile. Patients who used antibiotics, chlorhexidine, or fluoride mouthwashes within the last 2 weeks were excluded [20]. Additionally, children with special health care needs, with allergies to silver or any material included in the study, or those undergoing treatment for diseases affecting salivary flow were excluded. All eligible teeth per child were included.

During the recruitment phase, children were examined on a professional dental chair with operational light (Helios 3000 LED Dental Light, Pelton and Crane, Charlotte, NC, US) using a plane surface dental mirror and a CPI probe (Hu-Friedy, Chicago, IL, USA). Carious teeth were professionally cleaned with a prophy brush without any products and air-dried for 5 s. Decayed teeth were further inspected for caries activity using the blunt-ended probe according to the ICDAS lesion activity assessment criteria (LAA), and only active lesions were later included in the trial [19, 21]. At the baseline appointment, the participants were assessed for caries experience (dmft) using the World Health Organization (WHO) criteria [22].

#### Calibration

The principal investigator, NA, assessed caries lesion activity at baseline and follow-up appointments. NA was trained by two senior examiners (M.M.E.T and D.M.T) with over 20 years of experience in the field on the diagnosis of caries using the ICDAS criteria and assessment of caries activity using the ICDAS-LAA criteria [19, 21]. The investigator’s intra-examiner reliability was excellent (Kappa statistic = 0.93 and 0.91).

#### Randomization, allocation, and blinding

Participants were randomly and equally assigned in a 1:1 ratio to two groups using a computer-generated list of random numbers (https://www.randomlists.com/team-generator), and a trial-independent individual allocated the treatment. In the intervention group, the NSF formulation contained 256 μg/mL PEG-AgNP and 22,600 μg/mL fluoride in a deionized water-based solution. In the control group, 38% SDF (Advantage Arrest, Elevate Oral Care LLC., FL, US) was used. Blinding of the operator was not possible since the SDF solution has a bluish tint and stains demineralized hard tissues black unlike the NSF. However, the patients, microbiologist, and the biostatistician were blind to the intervention type.

#### Intervention

Children were asked to avoid eating two hours before the application of the intervention. All sample collection appointments were in the morning. During the appointment, caregivers completed the WHO questionnaire to assess children’s oral hygiene oral health practices (dental visits and sugar consumption), and their socioeconomic background (age, sex, and mother’s education) to enable the control of confounders [22]. Lesions were dried with a gentle stream of oil-free air for 5 s, then the tooth was partially isolated using a saliva ejector, petroleum jelly, and cotton rolls, and gross debris were removed with gauze. The baseline sample was collected by rubbing the caries lesion with a 2 mm wide habits (toothbrushing), micro brush (regular size Microbrush®, Henry Schein, Germany) for 4 s, then inserting the micro brush in 0.5 mL of saline in a sterile falcon test tube [18]. The test tubes were labeled with codes for participant, tooth, and time of sample collection. Within an hour of collection, all samples were transported at room temperature to the microbiology laboratories at the same university. No freezing was needed since processing started within one hour of harvesting the samples.

Following collection of the baseline sample, the interventions were applied. The isolated caries lesion was dried again. One drop of the solution per patient was dispensed into a plastic dappen dish. A disposable microbrush was dipped into the solution and any excess was dabbed on the walls of the dish. The agents were applied to the caries and the excess was removed using cotton swabs. The solution was left in contact with the tooth surface for one minute before children were allowed to close their mouths. Parents were asked to make sure that the children did not drink or eat for an hour [23]. After 24 h, the participants were contacted by phone to check if any adverse events had occurred.

#### Follow up examination

The follow-up, after one month, included sample collection using the same protocol and re-assessment of lesion activity using the ICDAS-LAA criteria where shiny and hard lesions were considered inactive [19, 21].

#### Microbiological procedure

Samples were vortexed for 30 s and serially diluted ten-folds with sterile saline. To detect *S. mutans* counts, ten microliter aliquots of each dilution were inoculated onto freshly prepared Mitis Salivarius agar plates (Difco Laboratories Inc, NJ, USA) and were anaerobically incubated in an incubator (redLINE incubator model RI 115-U, Binder GmbH, Tuttlingen, Germany) containing 10% CO_2_ at 37 °C for 72 h. Similarly, to detect *lactobacilli* counts, aliquots were inoculated onto Rogosa agar plates (Himedia Laboratories, Mumbai, India) and aerobically incubated for 48 h at 37 °C. Identification of *S. mutans* was confirmed by film morphology (gram-positive cocci), Catalase test (negative), and bile test (negative). *Lactobacilli* colonies appeared as white mucoid colonies on Rogosa agar and were confirmed using film morphology (long gram-positive rods, non-spore forming). Colonies grown on the plates which were enumerated, and the results were expressed as colony forming units per milliliter (CFU/mL) [24].

### Statistical analysis

Data were analyzed using IBM SPSS Statistics for Macintosh, Version 28.0. Armonk, NY: IBM Corp. Intention to treat analysis was used. Data were assessed for normality using the Kolmogorov–Smirnov test. Age, dmf, and the number of treated teeth were presented using mean and standard deviation. The study outcomes were the counts of *S. mutans* and *lactobacilli* after one month and the number of active caries lesions (dependent variables). Bacterial counts and percent reduction in bacteria were presented as median and interquartile range (IQR). Categorical variables were presented using frequency and percentage. The percent change in the bacterial count was calculated using the following equation:

$$Percent\,change = \frac{follow\,up\,count - baseline\,count}{{baseline\,count}} \times 100$$

Mann–Whitney U and Pearson Chi-Square tests were used to compare the log CFU and lesion activity, respectively, between the two groups. Changes in the bacterial log count and lesion activity after one month were assessed within each group using Wilcoxon Sign Rank and McNemar tests, respectively. Multilevel logistic regression analysis was used to assess the effects of the independent variables (interventions) that were introduced as fixed effects on reduction in *S. mutants* and *lactobacilli* counts (reduced, with negative percent reduction versus non reduced with zero or positive percent reduction) and the lesion activity (active versus non active), with controlling for the effect of confounders (age, gender, mother’s education, tooth type, brushing frequency, dental visits frequency, and sugar consumption, as well as the percent reduction in bacterial log counts for the model where reduction in lesion activity was the outcome). Adjusted odds ratios (AOR) and 95% confidence intervals (CIs) were calculated. Significance was set at *p* < 0.05.

## Results

### Synthesis, physical, and biological characterization of stabilized AgNPs

**Fig. 1:**
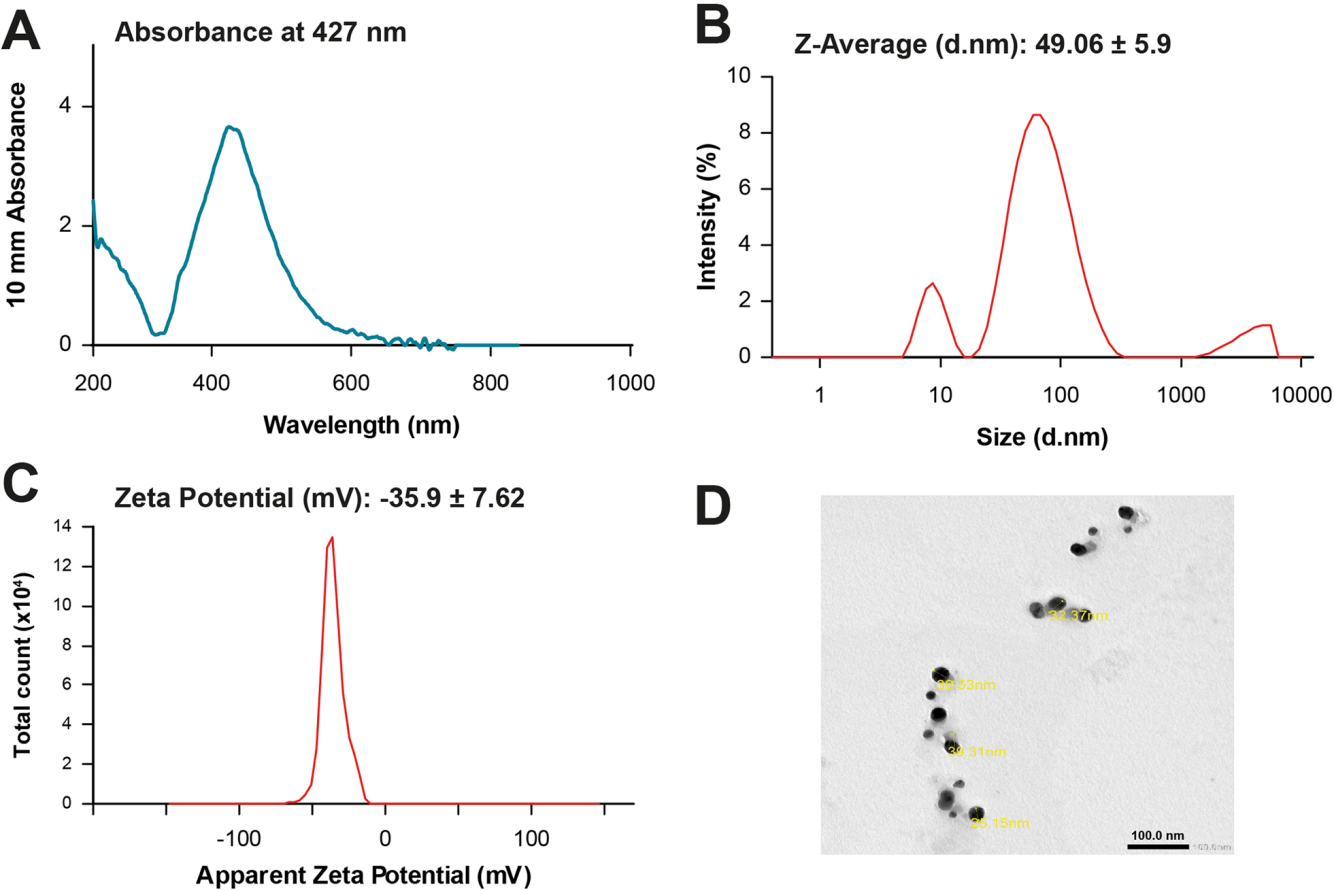
Optical and physiochemical characterization of PEG-AgNPs. **A** UV–Vis spectrophotometer showing a smooth regular peak with maximum absorbance at 427 nm, which is in accordance with the nanoparticles’ size range of 50–60 nm. **B** Dynamic light scattering technique showing an average size of 49 ± 5.9 nm, indicating and confirming the UV–Vis results. **C** Zeta potential revealing the synthesis of a highly stabilized silver nano population. **D** TEM image demonstrating the difference in intensities between AgNPs and their pegylated corona

PEG-AgNPs were successfully synthesized by the citrate reduction method. The UV–visible spectrophotometry showed a narrow peak with specific absorbance at 427 nm, reflecting the synthesis of nanoparticles with narrow size distribution. Dynamic light scattering revealed the formation of PEG-AgNP with an average size of 49.06 ± 5.9 nm and zeta potential of -35.9 ± 7.62 mV, indicating the stability of the formulated silver nanoparticles. TEM scans showed spherical nanoparticles with size ranging between 30 and 50 nm (Fig. 1).

**Fig. 2:**
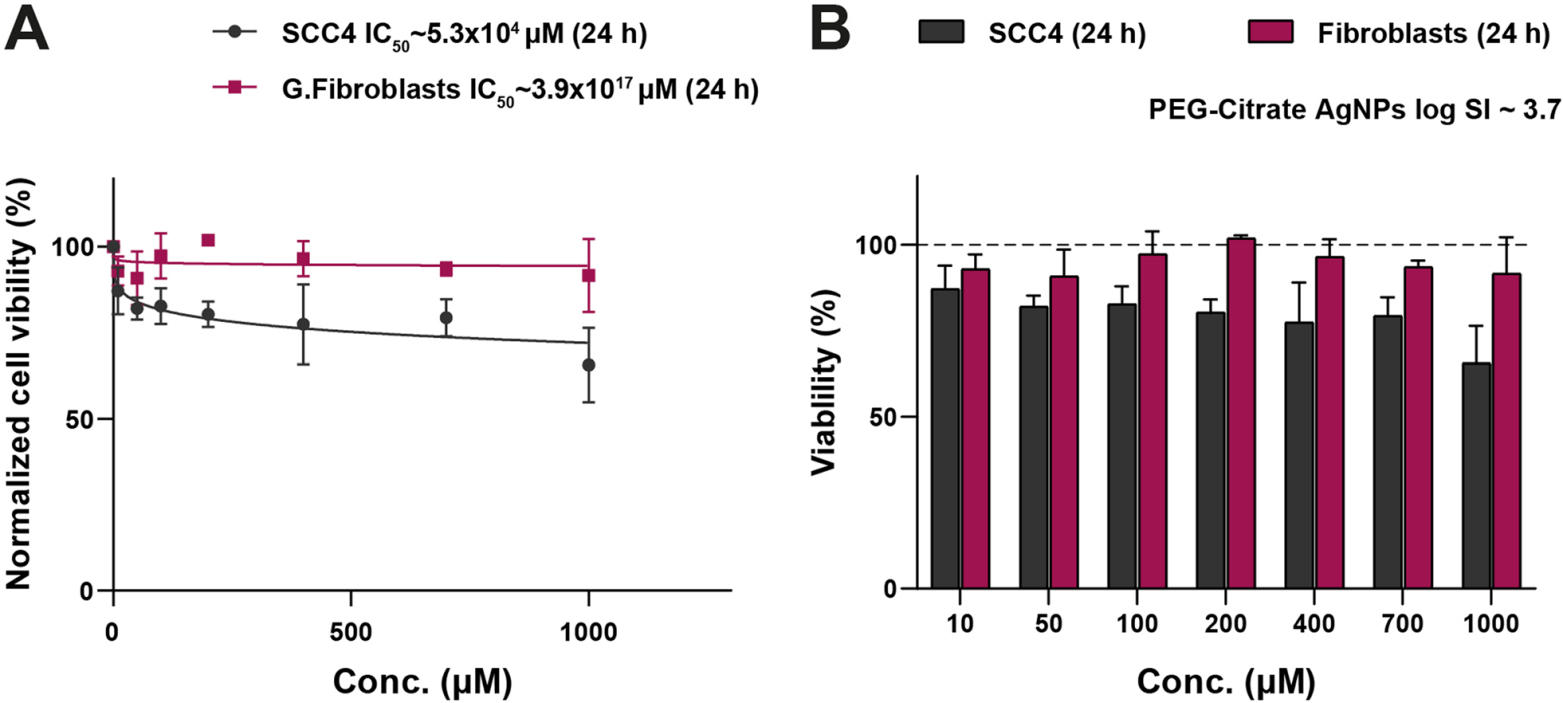
The biological behavior of the PEGylated AgNPs. **A** The dose dependent curves reveal the safety profile of PEG-AgNPs on normal gingival fibroblasts with a plateau curve as doses increase. Moreover, the minimal sensitization of the oral cancer cells to the PEG-AgNPs confirms their wide cytological biocompatibility. **B** A bar chart of the normal cell mean viability percentage (mean ± SD) versus cancerous cells reveals the high resistance of gingival fibroblasts to the PEG-AgNPs with high selectivity index (SI) pointed towards SCC4 cells

MTT cytotoxicity assay was conducted on oral SCC 4 and gingival fibroblast cell lines for 24 h. Non-linear regression analysis showed a plateau in the cytotoxicity curve indicating the nontoxic effect of PEG-AgNPs and a high safety profile, leaving cells almost 100% viable over time. The calculated SCC4 IC_50_ of PEG-AgNPs was 5.3 × 10^4^ µM which showed a 4.25-log fold increase upon dealing with gingival fibroblasts reaching 3.9 × 10^17^ μM as shown in Fig. 2. The MIC of non-fluoridated PEG-AgNPs on *S. mutans* was found to be 256 µg/mL.

### Clinical assessment of antibacterial and caries activity

The study sample included 130 teeth in 50 children. The mean ± SD age was 4.75 ± 0.76 years and most were males 26 (52%). There were no statistically significant differences at baseline between the groups in age, gender, and dmf (*p* > 0.05). In both groups, most children brushed their teeth less than once per day and had a high frequency of daily sugar consumption. Additionally, they reported visiting the dentist at least once in the last six months (Table 1).

**Table 1:** Sociodemographic profile and oral health behaviors of children in the study groups

	NSF 25 children, 71 teeth	SDF 25 children, 59 teeth	*P* value
Age: Mean (SD)	4.61 (0.61)	4.89 (0.88)	0.199
Gender: n (%)			
Male	14 (56%)	12 (48%)	0.571
Female	11 (44%)	13 (52%)	
dmft			
Mean (SD)	9.76 (4.31)	9.04 (3.71)	0.585
Number of treated teeth			
Mean (SD)	3.64 (1.60)	3.64 (1.93)	1.00
Tooth type: n (%)			
Anterior	29 (40.8%)	19 (32.2%)	0.309
Posterior	42 (59.2%)	40 (67.8%)	
Mother Education: n (%)			
Less than high school	14 (56%)	12 (48%)	0.577
High school and higher	11 (44%)	13 (52%)	
Frequency of brushing: n (%)			
Less than once daily	17 (68%)	14 (56%)	0.382
At least once daily	8 (32%)	11 (44%)	
Dental visits last 6 months: n (%)			
At least once	15 (60%)	19 (76%)	0.225
Less than once or never	10 (40%)	6 (24%)	
Sugar consumption: n (%)			
Less than once daily	10 (40%)	6 (24%)	0.225
At least once daily	15 (60%)	19 (76%)	

**Fig. 3:**
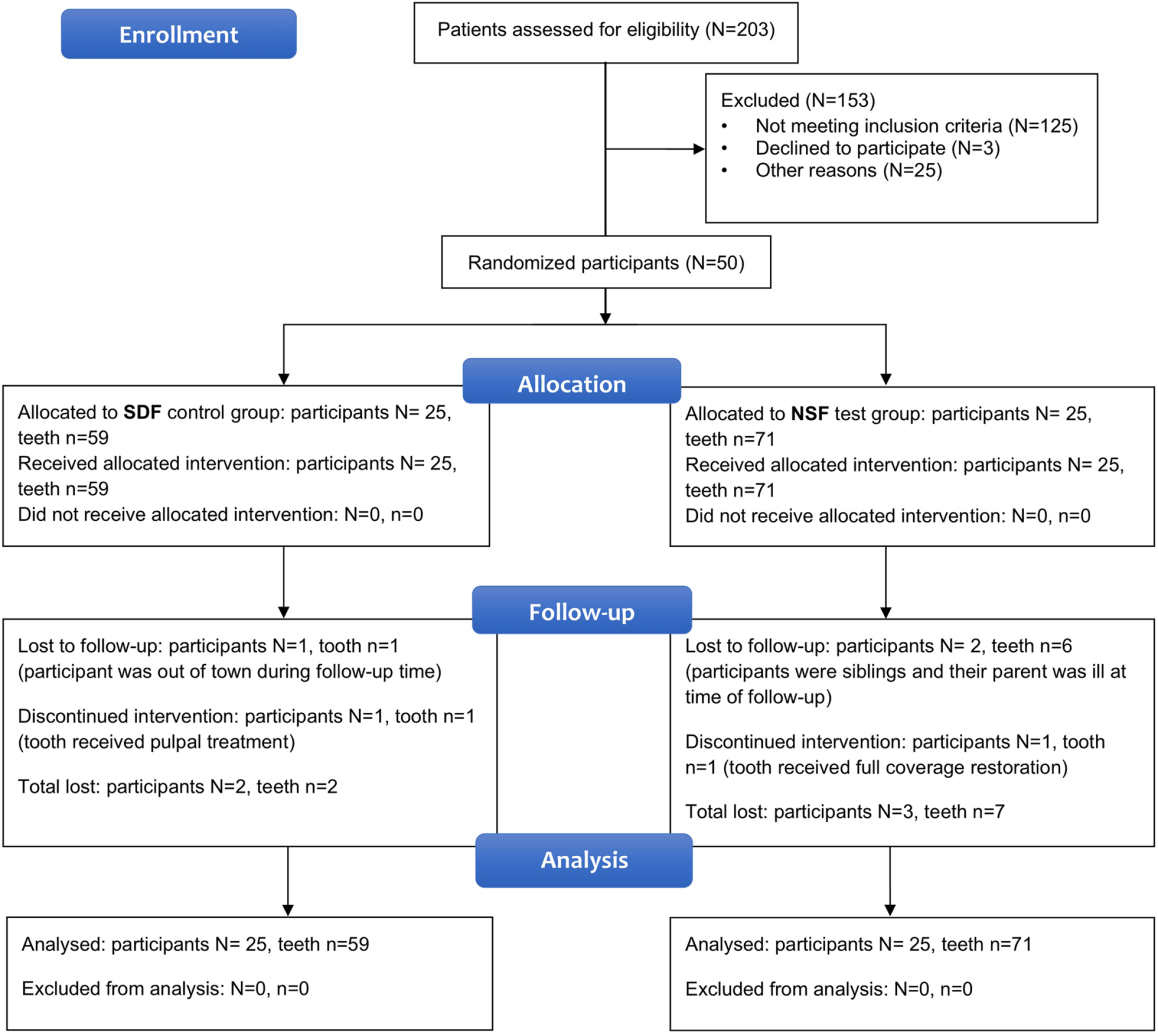
Study plan flowchart

NSF was applied to 71 teeth in 25 participants and SDF was applied to 59 teeth in 25 participants. There were 42 (59.2%) and 40 (67.8%) molar teeth in the NSF and SDF groups, respectively. After one month, nine teeth in five participants were lost to follow-up: seven in the NSF group and two in the SDF group. This is equivalent to 6.9% of teeth as shown in Fig. 3.

There was a significant reduction in the median log counts of CFU/mL of *S. mutans* and *lactobacilli* between baseline and after one month in both groups (*p* < 0.0001). The lesions treated with NSF showed a significantly greater median reduction in *S. mutans* than those treated with SDF (median (IQR) = 21.28% (100.00) and 10.46% (16.48), respectively, *p* = 0.002). The NSF group showed a higher median percent reduction in *lactobacilli* counts than SDF with no significant difference between groups (median (IQR) = 13.98% (100.00) and 6.02% (100.00), respectively, *p* = 0.094 (Table 2). When contacted after 24 h of the intervention, no participants reported any pain or signs of soft tissue injury after either intervention.

**Table 2:** Comparison between the study groups in *S. mutans* and *lactobacilli* counts in lesion samples at baseline and after one month

			NSF 25 children, 71 teeth	SDF 25 children, 59 teeth	*P* value
			Median (IQR)		
*S. mutans*	Baseline	Count	5.00 (8.00) × 10^4^	7.00 (7.00) × 10^4^	0.508
		Log_10_	4.70 (0.70)	4.85 (0.52)	
	1 month	Count	5.00 × 10^3^ (2.00 × 10^4^)	2.00 (2.00) × 10^4^	< 0.0001*
		Log_10_	3.70 (4.30)	4.30 (0.48)	
	*P* value		< 0.0001*	< 0.0001*	
	% Reduction		21.28 (100.00)	10.46 (16.48)	0.002*
*Lactobacilli*	Baseline	Count	5.00 (7.00) × 10^4^	3.00 (9.00) × 10^4^	0.814
		Log_10_	4.70 (0.90)	4.48 (1.00)	
	1 month	Count	0.00 (2.00) × 10^4^	9.00 × 10^3^ (5.00 × 10^4^)	0.026*
		Log_10_	0.00 (4.30)	3.95 (4.70)	
	*P* value		< 0.0001*	< 0.0001*	
	% Reduction		13.98 (100.00)	6.02 (100.00)0.094	

^*^Statistically significant difference at *P* value < 0.05

The lesions treated with NSF showed a significant reduction of 63.4% in active lesions, while those in the SDF group showed 64.4% inactive lesions (*p* < 0.0001) with no significant difference between groups in percent reduction (*p* = 0.903) as shown in Table 3.

**Table 3:** Comparison between the study groups in lesion activity after one month

		NSF 25 children, 71 teeth	SDF 25 children, 59 teeth	*P* value
After one month: n (%)	Active	26 (36.6%)	21 (35.6%)	0.903
	Inactive	45 (63.4%)	38 (64.4%)	
*P* value of reduction compared to baseline		< 0.0001*	< 0.0001*	

^*^Statistically significant difference at *P* value ≤ 0.05

The regression model showed that NSF may be better at reducing *S. mutans* counts (AOR 1.281, 95% CI 0.300–5.475) and *lactobacilli* counts (AOR 1.888, 95% CI 0.506–7.044) than SDF, although these differences were not statistically significant. The model also showed that caries treated with NSF were 1.35 times more likely to become inactive after one month than those treated with SDF (AOR 1.355, 95% CI 0.235–7.803) (Table 4).

**Table 4:** Multilevel binary logistic regression assessing the effect of NSF compared to SDF on reduction in *S. mutans* counts, reduction in *lactobacilli* counts, and change in lesion activity

	Reduction versus no reduction in *S. mutans* counts	Reduction versus no reduction in lactobacilli counts	Non active versus active lesions
AOR	1.281	1.888	1.355
95% CI	0.300, 5.475	0.506, 7.044	0.235, 7.803
*P* value	0.737	0.341	0.731
-2LL	611.537	591.885	630.235
% Correctly classified	88.5%	86.9%	90.8%
Model *P* value	0.669	0.868	0.719

Models are adjusted for age, gender, mother education, tooth type, brushing frequency, dental visits frequency, and sugar consumption. The model for lesion activity is additionally adjusted for the reduction in *S. mutans* and lactobacilli in lesions. -2LL: Log likelihood

## Discussion

This study demonstrated that NSF has considerable potency against cariogenic bacteria in dentin caries lesions, equal to that of SDF. Moreover, there was no difference in the cariostatic efficacy between both agents. Bivariate analysis revealed that NSF caused a significant reduction in *S. mutans* counts greater than that of SDF, but both agents had an equal and significant effect in reducing *lactobacilli* counts. However, in adjusted regression analysis, the differences between the two groups were not statistically significant. Thus, there is no support for rejecting the null hypotheses. To the best of our knowledge, there are no published clinical trials comparing the antibacterial effect of NSF and SDF on dentin caries lesions in primary teeth. This study fills a gap in the literature regarding the clinical efficacy of NSF on cariogenic bacteria and compares that effect to SDF.

The antibacterial activity of NSF is supported by multiple *in-vitro* studies. Yin et al. [25]*.* demonstrated that PEG-AgNP inhibit the growth of *S. mutans.* Targino et al. [15]*,* concluded that NSF is effective against *S. mutans* and that its effect is comparable to that of SDF. Despite being an etiologic agent in dental caries, the effect of NSF on *lactobacilli* has only been investigated in *in-vitro* studies. These studies showed that *lactobacilli* are susceptible to AgNPs, which is in line with the results of this trial [26, 27]. Clinical trials investigating the antibacterial effect of NSF are scarce. In agreement with the present results, Waikhom et al. [28] investigated the change in salivary *S. mutans* counts in children after one month of NSF application and reported 20% decrease bacterial in counts. Additionally, a pilot clinical trial by Freire et al. [8] revealed a significant decrease in the levels of *S. mutans* cultivated from the tooth biofilm after 24 h of NSF application.

The results showed bacterial reduction percent ranging from 6 to 21% in *S. mutans* and *lactobacilli* counts across groups (Table 2), which may insinuate limited clinical significance. However, this modest reduction was accompanied by 63% decrease in the number of active caries, which is the goal of the treatment. It is not clear to what extent these changes are interrelated, further studies are needed to better understand the relation between the change in bacterial counts and the corresponding change in caries activity.

The cariostatic potential and remineralizing effects of NSF have been successfully demonstrated in *in-vitro* studies and clinical trials [13, 16, 29]. Several clinical trials reported a decrease in the number of active caries after NSF treatment, ranging from 66 to 78% reduction after one year [9, 13, 14]. This is in line with the present results showing 63% reduction of active lesions after one month. The diminished magnitude of reduction may be attributed to the larger size of AgNPs used herein, since these studies used nanoparticles of smaller sizes, often below 10 nm. The effectiveness of AgNPs is known to be size-dependent, being inversely related to the nanoparticle size [30]. However, small sizes raise concerns about biocompatibility [31]. The concentration used in the current study was based on an MIC assessment, this was a lower AgNPs concentration than used previous studies. Furthermore, the unfavorable oral hygiene and dietary habits of the study participants (Table 1) may have influenced the compromised rate of caries arrest.

The silver and fluoride content are the prime factors contributing to the anti-caries activity of these agents. The 38% SDF used in this trial contained 256,721 to 289,565 ppm of ionic silver compared to 256 ppm of nanosilver in NSF, and 45,215 to 51,000 ppm of fluoride in SDF compared to 22,600 ppm in NSF [32]. Despite these substantial differences in concentrations between the two solutions, there were minor differences in their clinical performance regarding antibacterial and cariostatic efficacy, as is documented in the literature [13, 14, 16]. This can be attributed to the enhanced antibacterial activity of nanoparticles in comparison to larger compounds [30]. This fact can be profited on in MID. Through using NSF, the amounts of silver and fluoride that children are exposed to during treatment can be markedly reduced.

The AgNPs used herein had an average size of 49 nm which is commonly used in medical applications [25, 33]. The clinical use of this nanometer size is widely supported by scientific literature concluding that it poses no threat to safety [25, 30, 31, 33, 34]. Additionally, many published clinical trials investigated and followed-up NSF for up to one year after application, none of these trials reported any harms or side effects [8, 9, 13–16, 28]. Apart from formulating a safe NSF solution adhering to the published literature regarding clinically safe formulations, a cytotoxicity assay was conducted to further ascertain the safety of the nanoparticles for clinical application. The results of the assay confirmed that the PEG-AgNPs have a wide biosafety profile and a high selectivity index, leaving the normal cells almost 100% viable after application and incubation (Fig. 2). The minimal sensitization of the gingival fibroblasts to the PEG-AgNPs confirms their cytological biocompatibility.

One of the limitations of this clinical trial is that only two bacterial species were investigated. The Human Oral Microbe Identification using Next Generation Sequencing (HOMINGS) technology, based on 16S rRNA gene sequence analysis, has allowed the identification of uncultivable oral microbes [35]. Using this technology, *L. shahii, P. melaninogenica, V. dispar, Lep-totrichia HOT 498,* and *S. mutans* were found in children with at least 2 active carious teeth [36]. Similarly, an investigation into the microbiota of plaque samples harvested from adults with high daily sugar intake revealed that these individuals had a higher relative abundance of *S. sobrinus* and *P. melaninogenica* in comparison to individuals with low sugar intake [37]. Future studies are needed to assess the role of these bacteria in the caries process and the changes in the oral microbiome after NSF application.

Another limitation is the short follow-up period. Further studies with longer and more frequent follow-up intervals are needed to assess the long-term effects of NSF and certify that the results can remain comparable to those of SDF. Additionally, investigations into the optimization of this compound using different concentrations and to ensure its efficacy are needed before NSF can be used in minimally invasive caries management.

## Conclusion

The results of this study highlight the antibacterial efficacy and impact on caries activity of NSF and SDF. After one month, there was a significant reduction in *S. mutans* and *lactobacilli* counts in active dentin caries lesions, and two-thirds of the lesions became inactive. There were no differences between the two groups. Further research is needed with longer follow up periods to and multiple outcome assessors to confirm whether nanoparticles can be used to manage dental decay.

## Data Availability

The dataset generated and analyzed during the current study is available in the Synapse repository, https://www.synapse.org/#!Synapse:syn35283173/datasets/.
